# Deeper understandings of patterns of drinking among Aboriginal and Torres Strait Islander Australians: Informing policy and practice

**DOI:** 10.1002/hpja.696

**Published:** 2023-02-15

**Authors:** K. S. Kylie Lee, James H. Conigrave, Scott Wilson, Jimmy Perry, Noel Hayman, Tanya Chikritzhs, Robin Room, Teagan J. Weatherall, Catherine Zheng, Katherine M. Conigrave

**Affiliations:** ^1^ NHMRC Centre of Research Excellence in Indigenous Health and Alcohol, Discipline of Addiction Medicine, Faculty of Medicine and Health The University of Sydney Camperdown Australia; ^2^ The Edith Collins Centre (Translational Research in Alcohol Drugs and Toxicology), Sydney Local Health District Sydney Australia; ^3^ National Drug Research Institute, Faculty of Health Sciences Curtin University Perth Australia; ^4^ Burnet Institute Melbourne Australia; ^5^ Centre for Alcohol Policy Research La Trobe University Bundoora Australia; ^6^ Aboriginal Drug and Alcohol Council South Australia Underdale South Australia Australia; ^7^ Southern Queensland Centre of Excellence in Aboriginal and Torres Strait Islander Primary Health Care, Inala Australia; ^8^ School of Medicine Griffith University Australia; ^9^ School of Medicine University of Queensland Australia; ^10^ Centre for Social Research on Alcohol and Drugs, Department of Public Health Sciences Stockholm University Stockholm Sweden; ^11^ Drug Health Service, Royal Prince Alfred Hospital Camperdown Australia

## 1 BACKGROUND

It can be difficult to ask people how much alcohol they consume, and effectively communicate about ways to reduce alcohol‐related harm. Members of the public may not know the number of standard drinks that they usually consume, and the risks faced will vary according to their drinking pattern.^1^ For example, someone who consistently consumes 14 drinks a week over long spans of time is at increased risk of cancers.^2^ Whereas someone who sporadically drinks 10 drinks on single occasions is at greater risk of acute harms, such as physical injury or cardiac arrhythmia. We created and validated “The Grog Survey App” (“The App”) – an electronic tool to help Aboriginal and Torres Strait Islander peoples describe their drinking. This App also aims to help individuals better understand the risks associated with their drinking pattern, and gives them tailored feedback on their drinking. In this paper we discuss why and how the Grog Survey App was developed, and how the lessons learned from its use can inform health promotion and clinical practice. We consider how the App been used so far, and how it could be better leveraged for health promotion and to improve clinical workflows.

For Aboriginal and Torres Strait Islander peoples in Australia, and First Nations peoples in other similarly colonised countries, drinking patterns and associated risks may differ from the general population because of ongoing harms from colonisation.^3^ However, there is also great variation in how alcohol is consumed across and within Aboriginal and Torres Strait Islander communities.^4^ Communities vary in the proportions of people at long‐ and short‐term risk from drinking,^4^ in the types of alcoholic beverage typically consumed and types of drinking container. Information on local drinking culture and context is needed to enhance health promotion efforts.^1^ Accurate survey data about drinking can add valuable detail to what is learned from consultation.

However, accurately measuring self‐reported alcohol use is challenging and costly in any population.^5^ These challenges are amplified when surveying populations that are culturally distinct from majority populations,^6^ or who experience discrimination and stigma, which can make conversations about drinking threatening.^7^ Due to these challenges, policy makers have often relied on national estimates of drinking risk for Aboriginal and Torres Strait Islander Australians. However, these estimates may not reflect local drinking in many communities,^4^ and do not paint a sufficient picture of how alcohol is consumed locally (quantity per occasion, frequency, beverage type, drinking containers, setting).^5^ Health promotion materials that focus on types of alcohol that are rarely consumed in communities, or on patterns or contexts of drinking alcohol that are uncommon are unlikely be effective. Accurate and detailed measurement of drinking patterns is also important to ensure equitable and appropriate resource allocation for prevention, early intervention and treatment.^5^

## 2 RE‐THINKING HOW WE ASK ABOUT DRINKING

To address the challenge of collecting accurate, detailed and locally “granular” data on drinking patterns among Aboriginal and Torres Strait Islander Australians, we developed^8^ and validated^9^, ^10^ a tablet computer‐based population survey tool – “The Grog Survey App.” The ground‐up iterative process used to develop the App, prioritised perspectives of Aboriginal and Torres Strait Islander Australian health professionals.^8^ The resulting App is visual and interactive. The App was designed to replicate a conversational interviewing style that is used by experienced Aboriginal health professionals,^8^ and was recommended for interactions with Aboriginal and Torres Strait Islander individuals in national alcohol treatment guidelines.^11^ The App asks the respondents questions in plain English,^12^ and male and female voiceover options are available in both English and a First Nations Australian language (Pitjantjatjara).^8^ Images are used to help depict realistic pictures of people drinking.

The App is designed to be independently completed by participants (on an iPad with headphones) after set‐up by a research assistant. This makes the App a potentially cost‐effective tool compared to surveys that require interviewer administration. The App can operate “offline,” with data later pushed to a secure server when an internet connection is resumed. Accordingly, the App can be used in isolated settings where internet may not be available or reliable.

The choice of survey items on the App and their delivery was informed by consultation and research on Aboriginal and Torres Strait Islander ways of drinking.^1^, ^4^, ^8^ These approaches – now validated – provide insights to inform health promotion materials or conversations around alcohol use. For example, when the App asks the three items about alcohol consumption from the Alcohol Use Disorders Identification Test‐Consumption,^13^ it uses a pictorial modification of the questions about alcohol quantity. Commonly used alcohol types and drinking containers are used to display drinking thresholds, rather than asking about standard drinks. As intermittent drinking is common, and is often driven by social occasions such as Sorry Business (after deaths) or celebrations, the App does not assume regular drinking. Instead it asks about up to four recent drinking occasions in the last 12 months (a modified “Finnish” method).^13^ The distance between drinking occasions then provides a valid estimate of drinking compared to a clinical assessment.^9^

**FIGURE 1:**
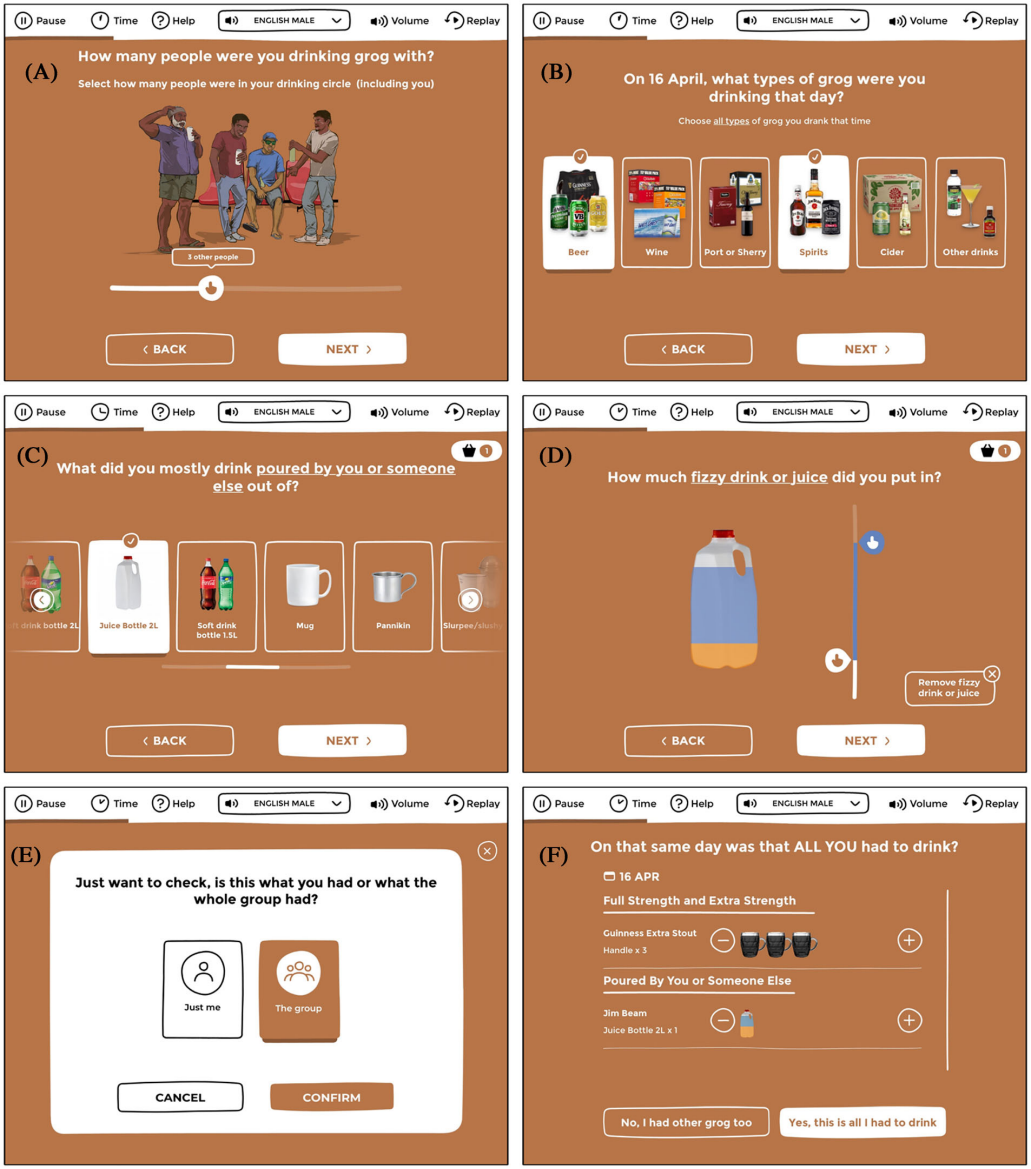
Screengrabs from the Grog App: An abridged user journey through “the Grog Shop.” After indicating when alcohol was last consumed in the previous 12 months, each person is asked how many people they were drinking with on the last drinking occasion (A). Each person then selects the types of alcohol consumed (B) and what containers they drank from (including standard and repurposed drinking vessels) (C). Sliders are used to indicate how full the container was with alcohol and any nonalcoholic mixers (D). If more than 7 Australian standard drinkers is consumed, the App checks if this was shared with a group or consumed by themselves (E). A summary is then presented of alcohol consumed per occasion; changes can be made to this drinking diary as necessary (F)

When describing recent drinking occasions, App users can select from a wide range of alcohol types (with realistic images) and container varieties (including repurposed containers such as empty soft drink bottles). Sliders allow users to indicate container fullness and number of containers consumed^8^ (Figure 1). For those who drink in a group where alcohol is shared (eg, pouring from a shared cask or bottle of wine, or selecting cans from a shared case of beer), the App allows users to describe their drinking as a share of how much the whole group drank.^8^

In keeping with this approach, health promotion materials around alcohol should not assume a regular drinking pattern, or that an individual can convert guidelines on alcohol use from standard drinks. As episodic drinking to intoxication (with risk of acute harms from alcohol) is more common that daily drinking to excess, the Australian guideline on keeping to a maximum of four standard drinks per occasion is particularly relevant. Converting this guideline^2^ into a culturally relevant form is important.

On the Grog Survey App, each individual receives a brief intervention via the App, tailored to their survey responses. This feedback is visual, and based on an adaptation of the brief intervention developed in a World Health Organization collaborative study.^14^ Guidelines on recommended drinking limits, are expressed visually, including guidance for when alcohol is shared. As well as receiving feedback on whether consumption is hazardous (ie, exceeds NHMRC recommended limits), the person also receives gentle feedback on whether they may be already experiencing “worries” from their drinking, in the form of either alcohol‐related harms, or symptoms of dependence (based on assessment of the three core ICD‐11 features of dependence).^15^ This has relevance to health promotion efforts – which, while aimed at preventing risky drinking, are typically delivered to whole communities, with a full spectrum of alcohol use.

## 3 USING THE APP TO IMPROVE HEALTH PROMOTION CAMPAIGNS

Aboriginal and Torres Strait Islander communities have successfully driven action to prevent or reduce harms from alcohol in many regions.^16^ These actions range from supply reduction, through to community development and health promotion,^17^ and harm reduction, such as night patrols.^16^ However, communities are typically not able to determine the success of their efforts by monitoring alcohol consumption. Instead, they monitor “downstream” events, such as major alcohol‐related harms. Accurate local assessment of drinking, using interactive and visual tools such as the Grog App, could help to shift the balance to enable communities and local services to take the lead in monitoring efforts to address drinking. This is instead of relying on outside research expertise.

Data from the App presents opportunities to tailor health promotion materials so that they are relevant, and compelling based on local drinking practices. For example, using The App, we found stark differences between drinking cultures in two communities – one urban and one remote Aboriginal community in South Australia.^18^ Respondents from the urban community typically consumed beer, spirits, and wine from a range of improvised containers – especially drinking glasses and mugs. Whereas the remote community largely consumed beer and pre‐mixed drinks from cans. Such differences mean that a single health promotion campaign would not likely be successful for use across both communities. In the remote community, a simple campaign about counting cans of drinks to monitor drinking may have been effective, whereas the urban community would require more complex education to help individuals understand how much alcohol they are consuming when using unstandardised drinking containers.

Creating such tailored campaigns involves time and costs, while the App may be able to play a direct and immediate role in health promotion campaigns. The App's detailed alcohol assessment combined with tailored brief intervention, appears to help users reflect on how much alcohol they drink.^10^ Aboriginal Australian research assistants reported that study participants spontaneously commented that the App “got them thinking” about their drinking.^10^ This suggests that the App could be an engaging and low‐cost health education tool in under‐resourced settings. This would be particularly useful in remote communities where there are typically fewer trained health professionals, and many of these come from a different language and cultural context to the communities they serve. Formal study of the extent to which use of the Grog Survey App could reduce risky drinking would be valuable.^19^

## 4 MONITORING AND RESPONDING TO RISKY DRINKING AND DEPENDENCE WITH THE APP

Substance use can change seasonally and over time; communities may not have single static risk profile.^20^, ^21^ Ongoing monitoring is essential to ensure communities receive extra support when needed – and wanted. On the other hand, funding can be reallocated to other communities at times when support is no longer required. Communities could use the App for this ongoing monitoring. In addition to estimating the proportion of people at long‐ and short‐ term risk, the App monitors prevalence of alcohol dependence.^22^ Beyond identifying whether a community needs support, the App can also help identify which population subgroups within communities are most at risk. For example, using data collected with the App, in one community we found that men and those with lower incomes were more likely to be dependent on alcohol.^22^ This information could be used to establish support groups for men, so they can connect with others and ensure their basic psychological needs are satisfied in safe ways.^23^

## 5 USING THE APP FOR FUTURE RESEARCH

In addition to monitoring and responding to local risk, the App can be used for a range of research purposes. We have designed the App to have flexible content. Default questions that are asked by the App can be removed, and new questions can be included (including voice over). This means that App content can be changed to meet local needs or to answer specific research questions. For example, we conducted research, using the App, to explore the links between finding alcohol need satisfying and risky drinking. We found that people who experience need satisfaction while drinking – who feel autonomous, connected to others and competent – are more likely to drink at risky levels, and become dependent.^24^ This finding suggests that creating safer ways to meet core psychological needs might be helpful in addressing risky drinking.

By allowing the App's content to be altered, research questions can be asked and answered that were not envisioned by the original development team. This will not only empower general researchers to use the App, but also will empower Aboriginal and Torres Strait Islander communities and health services who want to ask questions that meet their priorities.

## 6 WHAT'S NEXT FOR THE APP?

While the App has been proven to be a flexible and valid platform to conduct alcohol‐related research with Aboriginal and Torres Strait Islander communities, further enhancements could make it more useful. Many of the images in the App are specific to drinking alcohol, and cannot be changed. A content management system, could allow different survey items, visual content and voiceovers to be used for different projects. This would mean that the App could be completely overhauled to collect data about smoking, other drugs, or behaviours totally unrelated to substance use, in culturally appropriate ways. The App could also be used to support other priority populations, or be used in mainstream settings.

The App has the potential to be integrated into clinical systems. Currently, all data must be downloaded into CSV files to be accessed. However, there is no technical reasons why the App could not directly interface with clinical software systems. To do this, the App would need to be provided with a patient ID number or similar information. The patient could then fill out the survey while waiting for their appointment. On submitting the survey, the App could report findings back to the administrator's computer, which would automatically update their patient file with a report to be reviewed by a clinician. The clinician could then discuss the results with their patient without having to manually screen them, which is time consuming. Such a system would allow the App to increase clinical efficiency, while also ensuring people are regularly screened for risky drinking. Regular screening would help identify patients who need support, help monitor progress towards goals of cutting alcohol consumption, and flag when there are increases in drinking that might be affecting medication regimes.

The App could also play a role in population surveys. Due to the challenges of conducting culturally appropriate surveys, national surveys have often relied on small samples of Aboriginal and Torres Strait Islander peoples, from discrete geographical areas. Due to wide variations in drinking patterns, this may not always yield representative samples. Digital tools such as the Grog Survey App could be used in national surveys in multiple communities to enable more accurate estimates of drinking patterns.

## 7 CONCLUSION

The Grog Survey App uses digital technology to make it easier to ask individuals about the sensitive topic of alcohol use. The concept for the App was developed with Aboriginal Australian co‐investigators and health professionals. This promising tool^25^ allows description of detailed drinking patterns, and can inform how we ask about drinking in surveys and clinical settings. More broadly, the Grog App has the potential to provide fresh insights to inform policy, practice and community‐led action to reduce harms from alcohol. The Grog App is also likely to be relevant for other priority populations where episodic drinking, use of non‐standard drinking containers, and fear of discrimination and stigma are features of the drinking context.

## AUTHOR CONTRIBUTIONS

**KS Kylie Lee:** Co‐conceived the idea for the Grog App, conceived the idea for the commentary, investigator on the Grog App study, co‐led the manuscript writing, approved final manuscript. **James H Conigrave:** Led or supervised majority of Grog App analysis included in this commentary, co‐led the manuscript writing, approved final manuscript. **Scott Wilson:** Co‐conceived the idea for the Grog App, investigator on the Grog App study, reviewed drafts of the manuscript, approved final manuscript. **Jimmy Perry:** Investigator on the Grog App study, reviewed drafts of the manuscript approved final manuscript. **Noel Hayman:** Investigator on the Grog App study, reviewed drafts of the manuscript approved final manuscript. **Tanya Chikritzhs:** Investigator on the Grog App study, reviewed several drafts of the manuscript approved final manuscript. **Robin Room:** Investigator on the Grog App study, reviewed several drafts of the manuscript approved final manuscript. **Teagan J Weatherall:** Reviewed drafts of the manuscript, approved final manuscript. **Catherine Zheng:** Reviewed drafts of the manuscript, approved final manuscript. **Katherine M Conigrave:** Lead investigator on the Grog App study, reviewed several drafts of the manuscript.

## CONFLICT OF INTEREST

The authors declare no conflict of interest.

## Data Availability

The data that support the findings of this study are available on request from the corresponding author. The data are not publicly available due to privacy or ethical restrictions.
